# Ischemic damage may play an important role in spinal cord injury during dancing

**DOI:** 10.1038/s41393-020-0503-x

**Published:** 2020-06-19

**Authors:** An-Ni Tong, Jun-Wei Zhang, Hong-Jun Zhou, He-Hu Tang, Jin-Zhu Bai, Fang-Yong Wang, Zhen Lv, Shi-Zheng Chen, Shu-Jia Liu, Jie-Sheng Liu, Yi Hong

**Affiliations:** 1 Faculty of Rehabilitation Medicine, Capital Medical University, Beijing, China; 2 Department of Spine and Spinal Cord Surgery, China Rehabilitation Research Center, Beijing, China; 3 Department of Spinal Cord Injury Rehabilitation, China Rehabilitation Research Center, Beijing, China

**Keywords:** Spinal cord diseases, Disability

## Abstract

**Study design:**

Retrospective analysis.

**Setting:**

China Rehabilitation Research Center, Beijing, China.

**Objective:**

To explore possible mechanisms underlying spinal cord injury (SCI) in children caused by hyperextension of the spine while dancing.

**Methods:**

The clinical records of 88 children with SCI (mean age, 5.97 years; age range, 4–10 years) admitted to our hospital from January 1989 to October 2019 were retrospectively reviewed. Computed tomography and magnetic resonance imaging were performed on the day of injury. The time from injury to development of paralysis, as well as post-injury activities were surveyed, while abnormal patterns on images, the range of the involved vertebrae, and the extents of edema and atrophy were assessed.

**Results:**

Among the 88 patients, 6 (6.8%) were unable to move immediately after SCI, while paralysis occurred in 42, 23, and 17 patients at <30, 30–60, and >60 min after SCI, respectively. The neurological level of injury of 84 patients was between T4 and T12. On sagittal T2-weighted images (T2WIs), the longitudinal range of spinal cord edema was more than one vertebral body in 65 patients, while spinal cord atrophy below T8 was found in 40 patients. On axial T2WIs, although three patients had none, long T2 signals were found in the central gray matter of seven patients. Meanwhile, necrosis of the central area combined with the peripheral white matter was observed in 57 patients, while three patients had total involvement on a cross section.

**Conclusion:**

Ischemia-related damage, rather than direct trauma to the spinal cord, may play an important role in SCI due to spinal hyperextension during dancing.

## Introduction

**Fig. 1:**
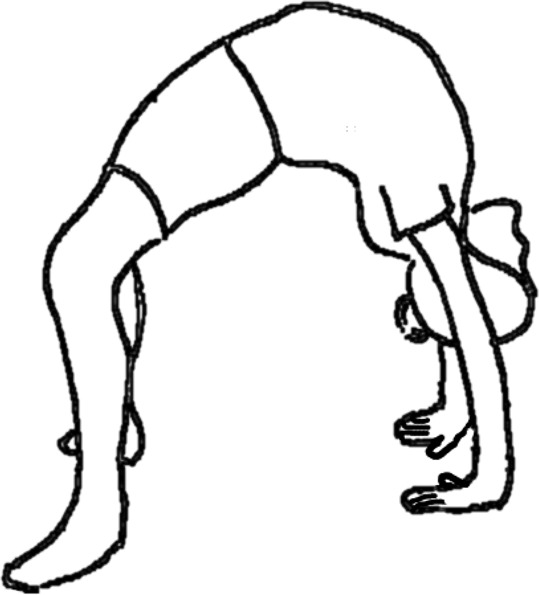
A representative image of spinal hyperextension: starting in a standing position and ending with hands touching the ground.

Spinal cord injury (SCI) can be devastating, especially for a child. Traffic accidents and falls are the main causes of SCI [1], although the incidence of sports injuries, including incomplete tetraplegia, have continued to gradually increase in recent years [2]. According to the literature, 5–69.5% of children with SCIs had no fracture or dislocation radiographically [1, 2]. SCI due to hyperextension of the spine while performing a back-bend during dance (Fig. 1) is a unique phenomenon in China and rarely reported abroad [3]. According to a domestic study, dance injury (50%) is the main cause of SCI in children, followed by car accidents (29.2%) and other factors (12.5%) [4].

Over the past two decades, an increasing number of girls have participated in after-school dance classes, which has resulted in an increase in the incidence of SCI. However, the consequence of such injuries to children and their families have not attracted the attention of the general public. Having had the opportunity to witness these sad stories, we felt obliged to study the mechanisms underlying SCI in order to prevent and mitigate such injuries, and to inform the general public about the potential dangers of practicing dance for children. Therefore, the aim of this retrospective analysis was to elucidate the underlying mechanisms in order to reduce the incidence of SCI.

## Methods

The clinical records, including injury details and radiographic data, of pediatric SCI patients (mean age, 5.97 years; age range, 4–10 years) admitted to the China Rehabilitation Research Center (Beijing, China) from January 1989 to October 2019 were retrospectively reviewed. All patients in the study cohort were female. The following three categories of data were collected from medical records, telephone follow-ups, and online questionnaire surveys: basic information (i.e., sex, age at time of injury, age at initiation of back-bend training, and history of fever or weakness), SCI details (i.e., starting position when performing a back-bend, use of protection, fall at time on injury, post-injury activities, and time between injury and paralysis onset), and diagnosis/classification of SCI. Here, the time of injury is defined as the initial onset of pain, numbness, or weakness. Computed tomography (CT), X-ray, or magnetic resonance imaging (MRI) was performed at 1, 7, 90, and >90 days after the injury.

The time from injury to paralysis onset was classified as <30, 30–60, or >60 min. All SCIs were graded in accordance with the International Standards for Neurological Classification of Spinal Cord Injury [5]. All SCIs were verified by CT, X-ray, or MRI with a focus on abnormal signals indicating ischemia and hemorrhage. The involved segments of each patient on sagittal T2-weighted images (T2WIs) were accumulated to create a distribution map of the range of spinal cord edema and atrophy. All abnormal intramedullary signals of spinal cord lesions on axial T2WIs were classified in accordance with the Brain and Spinal Injury Center (BASIC) scoring system [6].

## Results

**Fig. 2:**
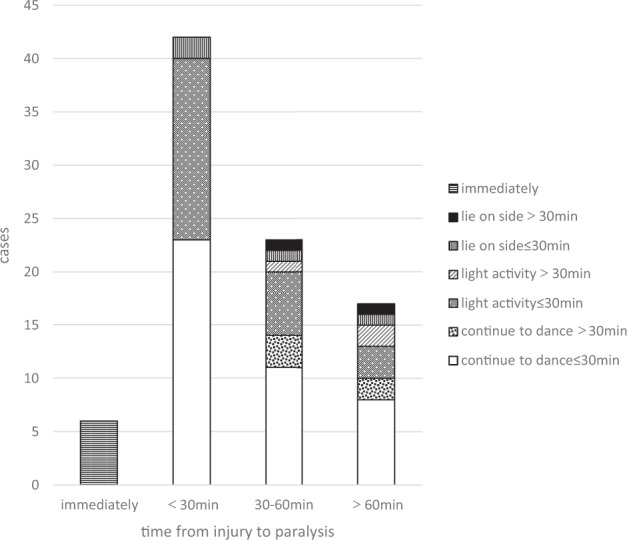
Post-SCI activity and time to the occurrence of paralysis. Six patients were unable to move immediately after sustaining a SCI. Four patients laid on their side for ≤30 min. Of these, two patients developed paralysis within 30 min, one in 30–60 min, and one in >60 min. Two patients laid on their side for >30 min. Of these, one patient developed paralysis in 30–60 min and one in >60 min. Of 26 patients who continued with light activities for ≤30 min, 17 developed paralysis within 30 min, six in 30–60 min, and three in >60 min. Of three patients who continued with light activities for >30 min, none developed paralysis within 30 min, one in 30–60 min, and two in >60 min. Of 42 patients who continued to dance for ≤30 min, 23 developed paralysis within 30 min, 11 in 30–60 min, and eight in >60 min. Of five patients who continued to dance for >30 min, none developed paralysis within 30 min, three in 30–60 min, and two in >60 min. Most of these patients continued to be active after injury for a duration of 30 min.

Of the 88 patients included for analysis, none had a history of fever or weakness before SCI. All patients reported spinal hyperextension at the time of injury and subsequently developed acute low-back pain as the first symptom, which was followed by weakness of the lower extremities and urinary/bowel dysfunction. Of these 88 patients, 84 (95.5%) had a neurological level of injury between T4 and T12. For most patients, the onset of paralysis occurred within 30 min after injury (Table 1). Post-injury activities were varied and the duration of most was about 30 min (Fig. 2).

**Table 1:** General information of 88 pediatric SCI patients.

	Cases (%)
Position when performing back-bends	
Standing	84 (95.5)
Kneeling	4 (4.5)
Location of paresthesia	
Low back	43 (48.9)
Lower extremities	35 (39.8)
Other	10 (11.4)
Symptom of paresthesia	
Pain	60 (68.2)
Numbness	23 (26.1)
Soreness	5 (5.7)
Time from injury to paralysis	
Immediately	6 (6.8)
<30 min	42 (47.7)
30–60 min	23 (26.1)
>60 min	17 (19.3)
ASIA impairment scale grade	
A	71 (80.7)
B	8 (9.1)
C	4 (4.5)
D	5 (5.7)
Level of injury	
T2	2 (2.3)
T3	2 (2.3)
T4	9 (10.2)
T5	2 (2.3)
T6	17 (19.3)
T7	6 (6.8)
T8	12 (13.6)
T9	8 (9.1)
T10	22 (25.0)
T11	4 (4.5)
T12	4 (4.5)

*ASIA* American Spinal Injury Association.

**Fig. 3:**
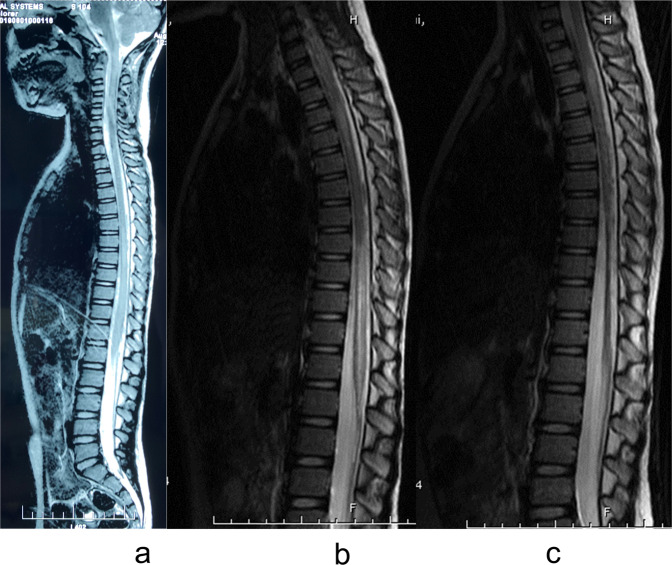
MRI of a 4-year-old girl with an injury to T4. **a** The day of injury with a slightly long T2 signal below T6. **b** One month later showing long signals at T3–4 and T8–L1 with a slightly long signal at T4–8. **c** Two months after injury showing a long signal at T3–4, a slightly long signal at T4–8, and spinal cord atrophy below T8.

**Fig. 4:**
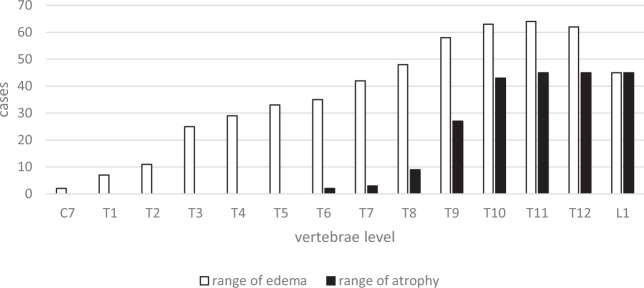
The distribution of edema and atrophy at the vertebral levels in 70 pediatric SCI patients. Data are presented as the cumulative range of edema and spinal cord atrophy. Edema occurred throughout the entire length of the thoracic spinal cord, but mostly involved the lower thoracic spine. The highest level of spinal cord atrophy occurred at T6 and was mostly concentrated at T8 and below.

**Fig. 5:**
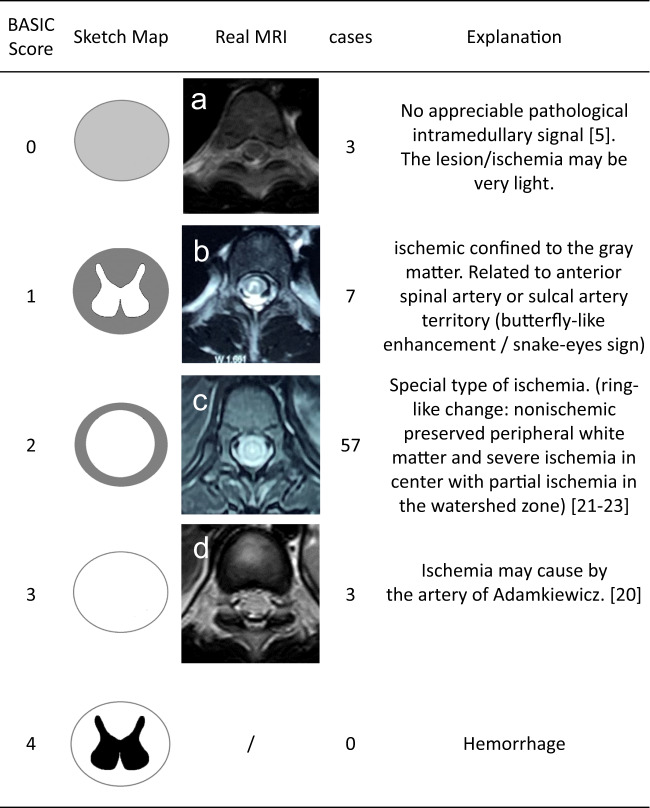
Classification of 70 pediatric SCI patients based on axial T2WIs. A schematic diagram of BASIC scores is shown in the “Sketch Map” column. BASIC score of 0: no abnormality; BASIC score of 1: involvement of the gray matter only; BASIC score of 2: involvement of both the central gray matter and adjacent white matter; BASIC score of 3: total involvement of the spinal cord; BASIC score of 4: involvement of the whole spinal cord with hemorrhage. **a** A 5-year-old girl with an injury at T8 with no appreciable abnormal signals. **b** A 10-year-old girl with an injury at T6 with “butterfly-like” enhancement of the gray matter, which is a typical sign of spinal cord ischemia. **c** A “ring-like” change of a 6-year-old girl with an injury to T10 mostly involving the central gray matter with some involvement of the adjacent white, but not the peripheral white matter. **d** Involvement of the whole spinal cord of a 6-year-old girl with injury to T11.

In the present study, the imaging data of 70 patients were collected and reviewed. For the other 18 patients, MRI data were not complete. No patient presented with a spinal fracture or dislocation on X-ray and CT images, although 67 patients had abnormal signals on T2WIs, which consisted of long T2 signals centering on the central canal of the spinal cord. The length of the long T2 signal was less than one vertebrae in two patients and more than one in 65. No patient had signs of significant hemorrhage on MRI (Fig. 3). Among these 67 patients, spinal cord edema most commonly involved the lower thoracic spine and lumbosacral medulla (Fig. 4). Of the 70 patients with imaging data, 45 had spinal cord atrophy, which was most commonly (40 patients) concentrated between T8 and the conus medullaris. Of these 45 patients, 30 (66.7%) developed atrophy within 90 days, including 4(8.9%) within 1 month, and 15 (33.3%) at more than 90 days. As to analysis of axial T2WIs, three patients had a BASIC score of 0, seven had a BASIC score of 1, 57 had a BASIC score of 2, three had a BASIC score of 3, and none had a BASIC score of 4 (Fig. 5).

## Discussion

Although most patients with SCI were young girls, one 14-year-old boy developed a SCI when performing a back-bend. However, since his teammate applied external force during the hyperextension, his thoracic vertebrae also fractured. So, this case was excluded.

The pathological mechanism of a thoracic SCI without fracture or dislocation remains unclear. Some researchers insist that the spinal cord is susceptible to traction injury, because it is guarded by the ribs during excessive flexion, but lacks protection against longitudinal traction, as confirmed by a cadaver study [7]. On the contrary, other researchers have suggested that ischemia is the primary mechanism of a thoracic SCI [8, 9], which can be explained by the anatomical features of the blood vessels surrounding the thoracic spine. The spinal cord is slender and the associated blood vessels have relatively small diameters with less collateral circulation. Therefore, any force that causes spasm or compression to a segmental artery can result in ischemic injury. Furthermore, it has been reported that the first symptom of this type of injury usually appears within 30 min and then progress rapidly, while segmental necrosis without evidence of ligament instability is often discovered during surgery or autopsy [1]. However, few studies have investigated the pathological mechanism of a SCI caused by dancing. A 2017 report by Ren et al. [10] of 12 patients of SCI among dancers speculated that the possible mechanism involves the nerves of the lower thoracic spine and lumbar perforation through the corresponding intervertebral foramina with little or no sliding, so that the distal end of the spinal cord is relatively anchored when performing a back-bend and that injury occurs when traction is beyond the tolerance of the spinal cord. In the present study, however, paresthesia of the lower back or lower extremities was found to be the first symptom, as 82 (93%) patients were able to move their lower extremities immediately after the injury and paralysis gradually developed within an hour. This delayed and progressive feature is consistent with previous reports on the mechanism underlying spinal cord ischemia [11]. In addition, if the spinal cord is directly injured by stretching, axonotmesis and subsequent paralysis typically occur immediately.

The anterior thoracic spinal cord has two or three arteries: less than that of the cervical and lumbosacral regions. The lower thoracic and lumbar spinal cord are mainly supplied by one Adamkiewicz artery, which originates from T9 to T12. Therefore, the thoracic spinal cord is more sensitive to ischemia. Once damage to the artery occurs, morphological changes occur to the blood vessels on the surface and within the spinal cord [12]. Longitudinal lengthening flattens and reduces the diameter of the spinal cord. As a result, the small arteries within the spinal cord simultaneously undergo the same morphological changes, especially the gray matter of the spinal cord and lateral column [13]. Novy et al. [14] found that nonsurgical spinal cord ischemia can be triggered by spinal hyperextension. Surfer’s myelopathy, which was defined by Thompson et al. [15] in 2004, seemed to be more similar to our patients, as none had a history of SCI and developed acute low-back pain due to prolonged duration in the prone position or standing up suddenly, which led to gradual aggravation of pain followed by weakness of the lower extremities within minutes to hours combined with urinary dysfunction. They speculated that the most likely cause was ischemia and that the possible mechanism was hyperextension of the spinal cord leading to infarction. However, there was an absence of evidence of embolism. All patients included in the present study had hyperextended the spinal cord at the time of injury and developed acute low-back pain as the first symptom, which was followed by weakness of the lower extremities and urinary/defecation dysfunction. The degree of extension is greater when performing a back-bend as compared with that when surfing. In addition, injury was concentrated to the middle and lower thoracic spinal cord in this study, in accordance with a report by Cheshire et al. [16].

A review of the MRI data of 70 patients found that the main abnormality in the first week after SCI was a long T2 signal around the center of the spinal cord, accompanied by swelling. In addition, sagittal images of 65 patients showed long T2W signals involving more than three vertebrae, most of which were located from T8–10 to the conus medullary, which sometimes included another long signal of the upper thoracic spine, mostly involving the T3–5 vertebrae corresponding to a poor blood supply. Two studies conducted by Bolton [17] and Zülch [18] pointed out that T4 and L1 were sensitive to ischemia, while Dommisse [19] found that the spinal canal is narrowest between T4 and T9, which results in an inadequate blood supply. Moreover, a 2013 imaging study conducted by Nakamoto et al. [20] of 23 patients with surfer’s myelopathy described “pencil-like” long T2 signals from the midthoracic spinal cord (T5–10) to the conus medullary. The abnormal range involved three to seven and a half vertebrae, which was accompanied by enlargement of the conus medullary, similar to the patients in the present study. More importantly, the late stage of ischemia was characterized by spinal cord atrophy. In the present study, spinal cord atrophy below the site of injury was found in 45 patients (64.3%) at more than 3 months after the injury. The levels of spinal cord edema during the early stage of injury and subsequent atrophy were concentrated between T8 and T10, which was the area of the spinal cord with a poor blood supply.

Characteristics of ischemia could also be found on axial T2WIs in the present study. SCIs of seven patients had a BASIC score of 1 with a typical “snake-eye” sign or “butterfly-like” gray matter enhancement. Fifty-seven patients had a BASIC score of 2, which involved the central gray matter and adjacent white matter, but not the peripheral white matter. These changes are consistent with the characteristics of spinal cord ischemia [21, 22]. In support of this view, Thompson [23] summarized the phenomenon of central necrosis and the involvement of the adjacent white matter, but not the peripheral white matter, and proposed that the gray matter is the most susceptible to ischemia, followed by the adjacent white matter, while the peripheral white matter is least susceptible. A 2005 imaging study conducted by Ishizawa et al. [22] described “ring-like” changes caused by ischemia, as further confirmed by Wang et al. [24], which were closely related to the anatomy of the blood vessels supplying the spinal cord. In this scenario, the anterior spinal artery fed the sulcus artery to supply the gray matter, as well as the lateral and base of the posterior white matter, while the peripheral white matter was supplied by the pial and coronary arteries. The intermediate region between the central and peripheral areas is a watershed that is supplied by the end of the two systems.

In conclusion, the pathological mechanism underlying SCI caused by performing a back-bend during dancing may be due to spinal cord ischemia caused by extreme dorsal extension. Notably, the incidence of SCI in China has continued to gradually increase over the past two decades, and has resulted in substantial medical and social burdens. Hence, it is urgent to prevent the occurrence and avoid aggravation of such injuries by emphasizing that hyperextension training during dancing should be done gradually with adequate protection. Also, if a dancer experiences a fall while performing a back-bend and then complains of being unwell, the individual should be advised to lie down and stop training in order to avoid further damage.

## Supplementary information

supplementary file

## Data Availability

See the supplementary file.
